# Data-Efficient EEG-Based Person Identification via Time-Interval-Based Multi-Positive Contrastive Learning

**DOI:** 10.3390/s26185891

**Published:** 2026-09-17

**Authors:** Xinghan Shao, Haixian Wang, Zhengping Wu

**Affiliations:** 1 Key Laboratory of Child Development and Learning Science of Ministry of Education, School of Biological Science & Medical Engineering, Southeast University, Nanjing 211189, China; shaoxinghan@seu.edu.cn; 2 School of Innovations, Sanjiang University, Nanjing 210012, China; 3 School of Electronic Science and Engineering, Nanjing University, Nanjing 210023, China

**Keywords:** electroencephalography, biometric, supervised contrastive learning, few-shot

## Abstract

Electroencephalography (EEG) is an emerging biometric trait because it is difficult to steal or forge and requires acquisition from living subjects. However, many EEG-based person identification methods rely on large enrollment datasets, increasing acquisition burden and limiting practical deployment. To address this issue, we propose a data-efficient person identification framework based on time-interval-based multi-positive contrastive learning (TIB-MPCL). For each one-second EEG segment, overlapping, adjacent, and non-adjacent positive samples are constructed to exploit temporal diversity. These samples are jointly optimized through contrastive learning, together with an alignment module that promotes intra-subject compactness and inter-subject separability. Experiments on the 109-subject PhysioNet dataset demonstrate strong performance under both sufficient- and limited-sample conditions. TIB-MPCL achieves an average accuracy of 99.05% using all available training samples and 83.86% using only 10 one-second samples per subject, with accuracies above 91% for motor execution and imagery. Cross-session experiments on SEED-IV further evaluate robustness to session-dependent variability, achieving an average accuracy of 83.79% when the test session is completely excluded from training.

## 1. Introduction

With the increasing demand for reliable identity verification in security-sensitive applications, biometric identification has become an important research topic. Compared with conventional biometric traits such as fingerprints, iris patterns, and facial characteristics, electroencephalography (EEG) has attracted growing attention as a secure biometric modality because it is generated by neural activity and is difficult to steal, duplicate, or forge [1,2,3]. Moreover, EEG signals are closely associated with active neural activity, which provides inherent liveness-related advantages and makes EEG-based person identification particularly suitable for high-security scenarios [4,5,6].

Although EEG-based person identification has shown promising performance in recent years, its practical deployment is still limited by the amount of enrollment data required from each subject. In a typical EEG-based biometric system, each user must provide a sufficient number of EEG samples during the registration stage so that the identification model can learn stable and discriminative identity-related features. However, EEG acquisition is more time-consuming and less convenient than the collection of many traditional biometric traits. Long registration sessions may reduce user acceptance and increase the difficulty of deploying EEG-based biometric systems in real-world environments [7]. Therefore, reducing the amount of required enrollment data while maintaining high identification accuracy is a key issue for practical EEG-based person identification.

Existing studies have developed various EEG-based person identification methods using handcrafted features and conventional machine learning classifiers, such as autoregressive coefficients, entropy-based features, frequency-band power features, mel-frequency cepstral coefficients, and functional connectivity features [4,8,9,10,11,12]. With the development of deep learning, models such as convolutional neural networks, recurrent neural networks, graph neural networks, autoencoders, and Transformer-based architectures have further improved the performance of EEG-based biometric recognition [1,13,14,15,16]. These methods can automatically learn discriminative representations from EEG signals and have achieved high accuracy on several benchmark datasets. However, most deep learning-based methods require a relatively large number of training samples for each subject. When only a few EEG samples are available, the learned representations may become unstable, and the model is more likely to suffer from overfitting and insufficient inter-subject discrimination [7,8].

Small-sample EEG-based person identification is therefore a challenging but important problem. Under limited enrollment conditions, the model must extract robust identity-related information from short-duration and scarce EEG samples. However, EEG signals are inherently nonstationary and easily affected by noise, changes in individual state, and acquisition conditions. As a result, samples from the same subject may exhibit considerable intra-subject variation, while samples from different subjects may contain overlapping patterns. This makes it difficult for conventional classification models to learn compact intra-subject representations and separable inter-subject decision boundaries when the number of training samples is limited [17].

Contrastive learning provides a promising solution for representation learning under limited-data conditions. By pulling similar samples closer and pushing dissimilar samples apart in the feature space, contrastive learning can enhance the discriminative ability of learned representations [18]. In particular, supervised contrastive learning uses class labels to construct positive and negative sample pairs, where samples belonging to the same class are treated as positives and samples from different classes are treated as negatives [19]. For EEG-based person identification, subject labels naturally provide supervision for constructing positive pairs. This allows the model to learn more compact identity representations from the same subject and more separable representations across different subjects. More importantly, multiple positive pairs can be constructed from a limited number of samples, which effectively increases the representation constraints without requiring additional EEG acquisition.

To address the problem of EEG-based person identification with limited enrollment samples, this study proposes a time-interval-based multi-positive contrastive learning algorithm, named TIB-MPCL. The proposed method is designed to learn discriminative identity-related representations from small-sample EEG data. Specifically, EEG time-domain features are first extracted from short EEG segments. Then, a time-interval-based multi-positive sample construction strategy is introduced. For each anchor EEG segment, three temporally related positive samples are generated according to their temporal distance from the anchor sample, including overlapping, adjacent, and non-adjacent samples. The three types of augmented samples are jointly incorporated into a supervised contrastive learning framework to provide multiple positive views for each anchor segment. In this way, the model can jointly exploit local temporal consistency, adjacent temporal relationships, and broader temporal diversity provided by non-adjacent EEG segments. Through the unified multi-positive contrastive objective, the proposed model enhances intra-subject compactness and inter-subject separability, thereby improving identification performance under limited training samples.

The proposed TIB-MPCL model is evaluated on an EEG dataset containing 109 subjects under multiple experimental conditions. Experimental results show that the proposed method achieves strong identification performance even when only a small number of samples are available for each subject. In particular, when only 10 one-second EEG samples per subject are used for enrollment, TIB-MPCL achieves an average identification accuracy of 83.86% across four paradigms, with accuracies above 91% in both the motor execution and motor imagery paradigms. These results demonstrate its effectiveness for small-sample EEG-based person identification.

The main contributions of this study are summarized as follows:

- A data-efficient EEG-based person identification framework, named TIB-MPCL, is proposed to reduce the dependence of EEG biometric systems on large-scale enrollment data. By combining AR-based temporal feature extraction with supervised contrastive learning, the proposed framework can learn discriminative identity-related representations from short and limited EEG samples.
- A time-interval-based multi-positive sample construction strategy is designed to exploit the temporal structure of EEG signals. Specifically, overlapping, adjacent, and non-adjacent positive sample pairs are constructed for each anchor sample to capture local temporal consistency, adjacent temporal relationships, and broader temporal diversity from non-adjacent segments. Together with the alignment module, this strategy enhances intra-subject compactness and inter-subject separability in the latent representation space.
- Extensive experiments are conducted on the 109-subject PhysioNet EEG dataset under multiple paradigms and sample-size settings. The results demonstrate that TIB-MPCL achieves highly competitive performance under sufficient-sample conditions and significantly outperforms existing methods under limited-sample conditions. In addition, authentication analysis based on ROC, AUC, EER, and score distributions, together with ablation experiments and reduced-electrode evaluations, further verifies the effectiveness of the proposed framework. To complement the within-session evaluation on PhysioNet, cross-session experiments are further conducted on the SEED-IV dataset, where EEG recordings from independent sessions acquired on different days are separated between training and testing, providing additional evidence of robustness to session-dependent variability.

## 2. Related Work

### 2.1. Small-Sample EEG Biometric Identification

EEG-based biometric identification has attracted increasing attention because EEG signals are generated by neural activity and contain subject-specific patterns that are difficult to steal, duplicate, or forge. Early EEG biometric studies mainly focused on verifying whether EEG signals contain stable and discriminative person-specific information. For example, Maiorana et al. investigated the permanence of EEG signals for biometric recognition, while Ruiz-Blondet et al. proposed the CEREBRE protocol for event-related potential-based biometric identification [20,21]. Subsequent studies further explored cognitive EEG neurodynamics, functional connectivity, and task-independent person-specific EEG signatures for biometric identification [22,23,24]. Recent deep learning-based EEG biometric methods have also considered security and privacy issues, such as cancellable EEG biometric representations [25]. Although these studies demonstrate the feasibility of EEG-based identification, most existing methods still require sufficient enrollment samples from each subject to learn stable identity representations, which increases the burden of EEG acquisition and limits practical deployment.

The amount of training data required for EEG biometric recognition has recently begun to receive specific attention. Gómez-Tapia et al. investigated the minimal amount of EEG data required for task-dependent EEG-based person identification and showed that feature representations such as power spectral density (PSD) and wavelet energy can substantially reduce the required training duration [17]. However, their experiments were conducted on the DEAP dataset, which contains 32 participants and affective EEG recordings, making the identification scale relatively limited. In contrast, this study evaluates small-sample EEG identification on the PhysioNet EEG Motor Movement/Imagery dataset with 109 subjects, where the larger subject population increases inter-subject similarity and makes discriminative representation learning more challenging. Therefore, reducing the number of enrollment samples while maintaining high identification accuracy remains an important problem for practical EEG biometric systems. Few-shot EEG studies further show that limited labeled data, noise, and inter- and intra-subject variability can weaken the generalization ability of traditional machine learning and deep learning models [26]. Under limited enrollment conditions, EEG biometric models may overfit scarce subject-specific samples, resulting in unstable intra-subject representations and insufficient inter-subject separability. Therefore, data-efficient representation learning is necessary for small-sample EEG biometric identification.

### 2.2. EEG Data Augmentation and Enrollment Efficiency

Data augmentation is an effective strategy for alleviating the shortage of EEG training data without requiring additional signal acquisition. In EEG deep learning, commonly used augmentation methods include noise addition, sliding-window segmentation, temporal cropping, signal recombination, channel-level recombination, feature transformation, and generative models [27]. These methods increase the diversity of training samples and reduce the dependence on long recording sessions. For EEG biometric identification, this is particularly important because the enrollment process directly affects user convenience. If a system requires a large number of EEG samples from each new user, the registration time becomes long and the practical usability of the biometric system is reduced.

Existing studies have shown that data augmentation can be especially useful when EEG data are limited. Rommel et al. systematically compared multiple EEG augmentation strategies and showed that appropriate augmentation can significantly improve model performance in low-data regimes [28]. In EEG identity recognition, Hu et al. proposed a spatial–temporal generative adversarial network to generate additional EEG samples for brainprint recognition [29]. Their method improves identity recognition performance by generating EEG data that preserve temporal and spatial characteristics. However, GAN-based augmentation usually requires an additional generative model and may introduce synthetic samples whose identity-related reliability is difficult to guarantee. Moreover, existing augmentation methods rarely consider how to construct reliable positive samples from very limited enrollment EEG for contrastive representation learning.

Therefore, for enrollment-efficient EEG biometric identification, augmentation should not only increase the number of training samples but also preserve subject-specific information. Instead of relying on complex generative models, this study constructs multiple positive samples from limited enrollment EEG by using time-interval-based segmentation. Overlapping and temporally adjacent segments provide locally consistent views of the same subject, while non-adjacent segments introduce additional intra-subject diversity. In addition, this study uses AR features to characterize the temporal dynamics of EEG signals, enabling the model to capture subject-specific temporal patterns from short EEG segments. This strategy enriches the training data under small-sample conditions and helps the model learn more robust identity-related representations without increasing the actual enrollment time.

### 2.3. Supervised Contrastive Learning for Limited EEG Samples

Contrastive learning has become an effective representation learning strategy by encouraging similar samples to be close and dissimilar samples to be separated in the embedding space. Supervised contrastive learning further extends this idea by using class labels to define positive and negative samples, where samples from the same class are pulled together, and samples from different classes are pushed apart [30]. Compared with conventional cross-entropy loss, supervised contrastive learning directly optimizes the structure of the feature space and can improve intra-class compactness and inter-class separability. This property is particularly suitable for EEG biometric identification, where subject identities naturally provide class labels and the learned representation should be compact within each subject while remaining discriminative across different subjects.

Recent studies have introduced contrastive learning into EEG and biosignal representation learning to address limited labels, noisy signals, and subject variability. Mohsenvand et al. proposed a contrastive representation learning framework for EEG classification by extending SimCLR to EEG time-series data and using channel recombination to increase the number of training samples [31]. Cheng et al. proposed subject-aware contrastive learning for biosignals and showed that subject information can be used to improve representation learning under limited-subject and noisy-label conditions [32]. Yang et al. further proposed ContraWR for self-supervised EEG representation learning, indicating that the construction of negative samples can strongly affect the quality of contrastive EEG representations [33]. These studies demonstrate the potential of contrastive learning for EEG representation learning, but most of them focus on general EEG classification, self-supervised pretraining, or cross-subject modeling rather than small-sample EEG biometric identification.

For limited-sample EEG biometric identification, a key challenge is that only a few enrollment samples are available for each subject, which restricts the number and diversity of positive pairs. Simply treating original samples from the same subject as positives may be insufficient to learn robust identity-related representations. To overcome this limitation, this study combines supervised contrastive learning with time-interval-based multi-positive sample construction. By generating overlapping, adjacent, and non-adjacent positive samples from limited EEG recordings, the proposed method provides richer positive constraints for each anchor sample. This encourages the model to learn compact intra-subject representations and maintain clear inter-subject boundaries, thereby improving identification performance under limited enrollment conditions.

## 3. Methodology

**Figure 1:**
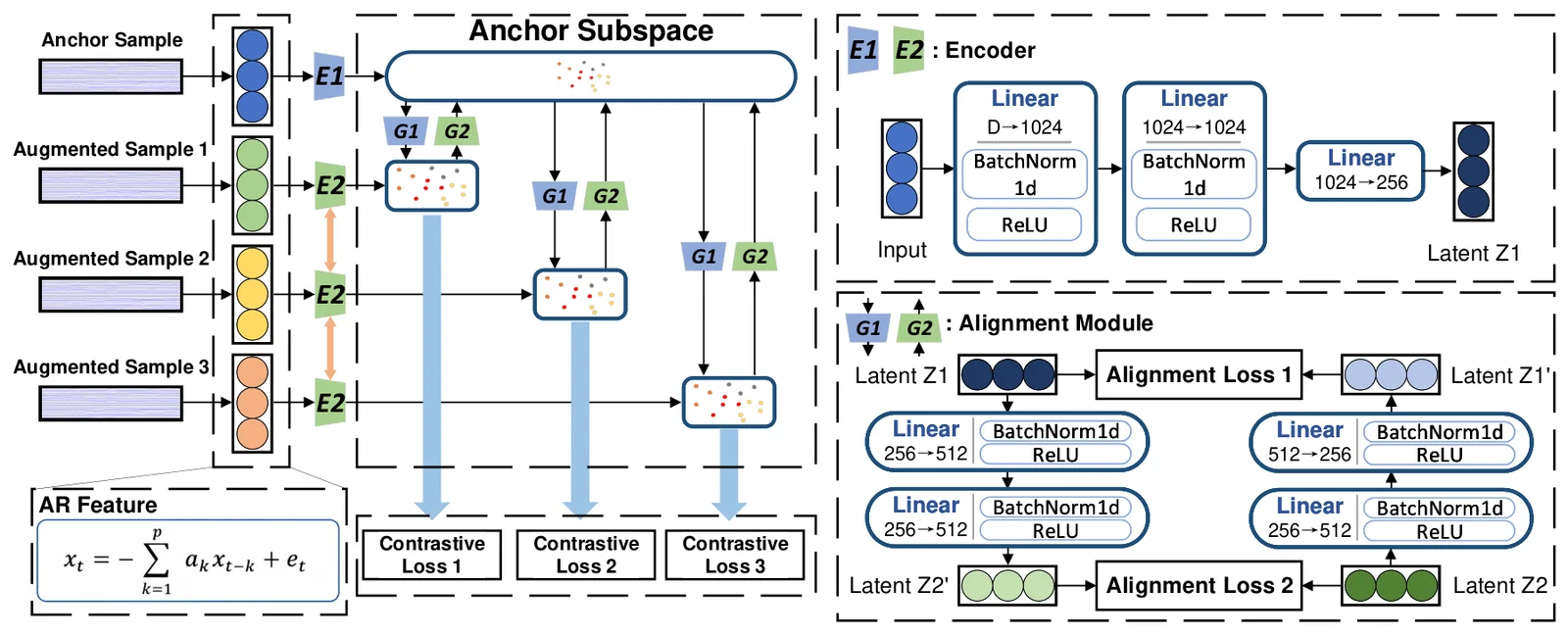
Overview of the architecture of TIB-MPCL.

Figure 1 illustrates the framework of the TIB-MPCL algorithm, which consists of a time-domain augmentation module, a feature extraction module, an encoder module, an alignment module, and a contrastive loss module.

### 3.1. Dataset and Processing

The PhysioNet dataset [34] is a publicly available benchmark dataset widely used in studies on motor imagery and EEG-based biometric identification. The dataset was acquired using the BCI2000 system and contains EEG recordings from 109 subjects, each of whom completed six experimental paradigms. These paradigms can be broadly divided into two resting-state tasks and four motor execution or motor imagery tasks. The resting-state tasks include eyes-open and eyes-closed resting states. The motor execution tasks require subjects to open and close the corresponding fist according to cues presented on the left or right side of the screen, or to perform bilateral fist or foot movements according to cues presented at the top or bottom of the screen. In contrast, the motor imagery tasks require subjects to imagine performing the corresponding movements under the same cue conditions. The two resting-state tasks were each recorded in one run lasting 1 min, whereas the other four tasks were each recorded in three runs lasting 2 min, resulting in a total of 14 runs for each subject. The raw EEG signals were recorded from 64 electrodes arranged according to the international 10–10 system, with a sampling frequency of 160 Hz. In this study, the original six paradigms were further grouped into four states: eyes-open resting state (EO), eyes-closed resting state (EC), physical motor execution (PHY, including Tasks 1 and 3), and motor imagery (IMA, including Tasks 2 and 4).

The SEED-IV dataset [35] is a widely used benchmark for EEG-based emotion recognition and cross-session evaluation. It was collected by the Brain-like Computing and Machine Intelligence (BCMI) Laboratory at Shanghai Jiao Tong University and contains EEG recordings from 15 subjects, each participating in three experimental sessions conducted on different days, with an interval of at least two days between consecutive sessions. Each session consisted of 24 trials, with six trials corresponding to each of four emotional states: happy, sad, fear, and neutral. The stimuli used in the three sessions were completely different, introducing additional variability across recording sessions. During each trial, participants watched an emotional film clip lasting approximately 2 min, preceded by a 5-s start cue and followed by a 45-s self-assessment period. EEG signals were recorded from 62 channels according to the international 10–20 system using an ESI NeuroScan system at an original sampling rate of 1000 Hz. In this study, SEED-IV is used to evaluate cross-session generalization, since recordings from the same subjects were acquired on different days and under different emotional stimuli, providing a challenging setting for assessing robustness to session-dependent variability.

Before segmenting the continuous EEG signals into individual samples, the raw EEG data were preprocessed to improve signal quality and reduce inter-channel variability. A bandpass filter was first applied to retain frequency components within 0.5–40 Hz. For the PhysioNet dataset, the continuous EEG recordings of each subject and each state were chronologically divided into training and test sets at a ratio of 8:2. ICA was then performed independently on the training and test portions to attenuate artifacts, such that the ICA decomposition estimated from the test data was not used in the preprocessing of the training data, and vice versa. For the SEED-IV dataset, ICA was performed independently for each subject-session recording. For the held-out target session, the ICA decomposition was estimated exclusively from the EEG data of that subject-session recording, independently of the source sessions. Thus, the target session did not contribute to the ICA decomposition of the source sessions. In each cross-session configuration, the two source sessions were used for training, while the held-out target session was processed independently and used only for testing. Finally, z-score normalization was performed for each channel, with the mean and standard deviation estimated exclusively from the training data and subsequently applied to both the training and test data.

(1) $${\hat{x}}_{s,c}=\frac{x_{s,c}-{\bar{x}}_{c}}{\sigma_{c}},$$

where *s* and *c* indicate the sampling point and EEG channel, respectively. $x_{s,c}$ denotes the signal value at sampling point *s* on channel *c*, while ${\bar{x}}_{c}$ and $\sigma_{c}$ represent the mean and standard deviation of the samples from channel *c*, respectively.

### 3.2. Time-Domain Augmentation

**Figure 2:**
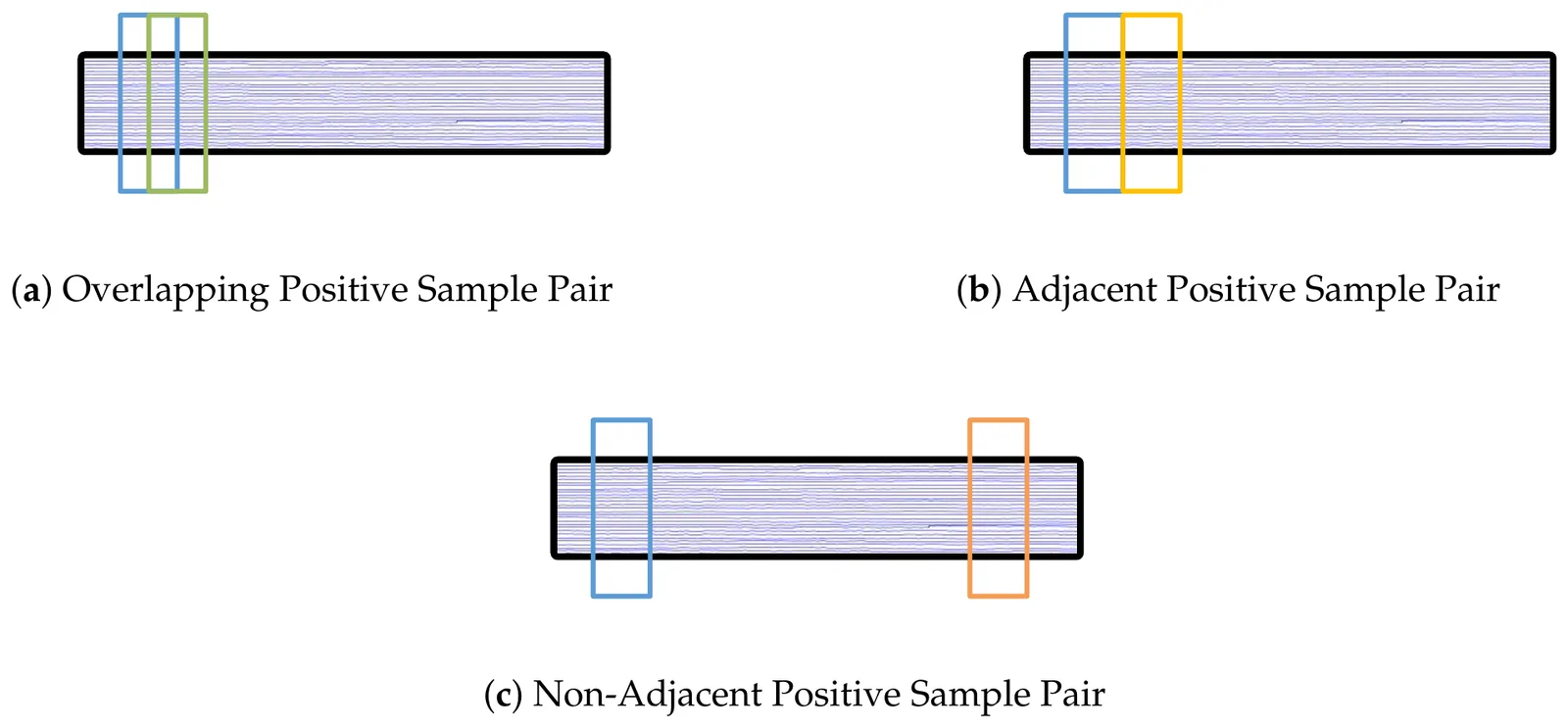
Time-domain augmentation. The blue, green, yellow, and orange segments denote the anchor sample, overlapping sample, adjacent sample, and non-adjacent sample, respectively.

EEG signals vary rapidly over short time intervals and exhibit strong non-stationary characteristics. To effectively account for the temporal properties of EEG signals, this study performs time-domain processing as follows. First, a 1-s EEG segment is selected as the anchor sample. Then, within the contextual EEG signal, 1-s signal segments with different temporal intervals from the anchor sample are captured as positive samples of the anchor sample. In this study, three time-domain augmentation strategies are adopted to construct positive sample pairs for contrastive learning, as shown in Figure 2: (1) Overlapping augmentation, where a 1-s segment overlapping with the anchor sample by 0.5 s is selected; (2) Adjacent augmentation, where a non-overlapping 1-s segment adjacent to the anchor sample is selected; and (3) Non-adjacent augmentation, where a 1-s segment with an interval of at least 2 s from the anchor sample is randomly selected within the EEG segment containing the anchor sample. Finally, the corresponding time-domain feature vectors of the anchor sample and its positive samples are extracted, respectively, and sequentially used as positive sample pairs input into the contrastive learning model.

### 3.3. AR Feature Extraction

To ensure consistency with the proposed time-domain augmentation strategy and to further characterize the temporal dynamics of EEG signals, AR-based temporal feature extraction was performed in this study. For each EEG channel, autoregressive (AR) modeling was employed to characterize the temporal dependency of the signal. Given a discrete EEG signal x(t), the *p*-order AR model can be expressed as

(2) $$x\left(t\right)=-\sum_{i=1}^{p}a_{i}x\left(t-i\right)+e\left(t\right),$$

where $a_{i}$ denotes the AR coefficient, *p* is the model order, and e(t) represents the prediction error or residual term. In this study, the model order was set to p=6. For each of the 64 EEG channels, the AR parameters were estimated using the Yule–Walker method. The estimated parameter vector was given as $\left[1,a_{1},a_{2},\ldots ,a_{6}\right]$, where the leading constant term was discarded, and the remaining six coefficients were retained as channel-level temporal features. Finally, the AR coefficients extracted from all EEG channels were concatenated to construct the final AR feature representation. Consequently, a 384-dimensional AR feature vector was obtained for each PhysioNet EEG sample. For SEED-IV, the same procedure was applied to its 62 EEG channels, resulting in a 372-dimensional feature vector. Accordingly, the input dimension of the encoder was set to 384 and 372 for PhysioNet and SEED-IV, respectively.

### 3.4. Encoder Module

The proposed data augmentation module constructs three types of positive samples for each anchor sample. Given a mini-batch of anchor samples denoted as $X={\left\{x^{\left(i\right)}\right\}}_{i=1}^{N}$, three augmented sample sets are generated, including the overlapping samples: $X_{O}={\left\{x_{O}^{\left(i\right)}\right\}}_{i=1}^{N}$, the adjacent samples $X_{A}={\left\{x_{A}^{\left(i\right)}\right\}}_{i=1}^{N}$, and the non-adjacent samples $X_{NA}={\left\{x_{NA}^{\left(i\right)}\right\}}_{i=1}^{N}$. Here, *N* denotes the number of anchor samples in a mini-batch, and *i* is the sample index.

To obtain temporally robust and paradigm-aligned representations, the anchor samples and their augmented counterparts are fed into two encoders with identical architectures, denoted as $E_{1}$ and $E_{2}$. Specifically, the anchor samples are encoded by $E_{1}$, while the three augmented views are encoded by $E_{2}$:

(3) $$z^{\left(i\right)}=E_{1}\left(x^{\left(i\right)}\right),$$

(4) $$z_{O}^{\left(i\right)}=E_{2}\left(x_{O}^{\left(i\right)}\right),$$

(5) $$z_{A}^{\left(i\right)}=E_{2}\left(x_{A}^{\left(i\right)}\right),$$

(6) $$z_{NA}^{\left(i\right)}=E_{2}\left(x_{NA}^{\left(i\right)}\right),$$

Both $E_{1}$ and $E_{2}$ adopt the same network configuration, i.e., $\left[D_{\mathrm{input}},1024,1024,256\right]$. Each hidden layer is followed by a batch normalization layer and a nonlinear activation function, ReLU.

### 3.5. Contrastive Loss Module

In this subsection, the overlap-based augmentation branch is used as an example to describe the construction of the contrastive objective. After feature encoding, the latent representations of the anchor samples and their corresponding overlapping augmented samples are concatenated into a single embedding set:

(7) $$Z=\{z^{\left(1\right)},z_{O}^{\left(1\right)},z^{\left(2\right)},z_{O}^{\left(2\right)},\ldots ,z^{\left(N\right)},z_{O}^{\left(N\right)}\}.$$

Each embedding vector is then normalized using the $L_{2}$ norm before similarity calculation:

(8) $${∥Z_{i}∥}_{2}=1,\;i=1,2,\ldots ,2N.$$

Based on the normalized embeddings, the similarity between the *i*-th and *j*-th samples is computed by the temperature-scaled cosine similarity:

(9) $$s_{ij}=\frac{Z_{i}^{⊤}Z_{j}}{\tau },\;i,j\in \left\{1,2,\ldots ,2N\right\},$$

where τ>0 is the temperature coefficient used to adjust the concentration of the similarity distribution.

During optimization, self-comparison is removed to prevent a sample from being treated as its own contrastive counterpart. Let I[·] denote the indicator function. The probability that $Z_{j}$ is selected as a positive sample for $Z_{i}$ is defined as

(10) $$p_{ij}=\frac{\exp\left(s_{ij}\right)I\left[i\neq j\right]}{\sum_{k=1}^{2N}I\left[k\neq i\right]\exp\left(s_{ik}\right)}.$$

For each embedding $Z_{i}$, its positive sample set is determined according to the class labels:

$$P\left(i\right)=\left\{j|j\neq i,\;y_{j}=y_{i}\right\}.$$

Here, $y_{i}$ and $y_{j}$ denote the labels of the *i*-th and *j*-th samples, respectively.

The overlap-based supervised contrastive loss is therefore formulated as

(11) $$L_{O}=-\frac{1}{2N}\sum_{i=1}^{2N}\frac{1}{|P(i)|+\epsilon }\sum_{j\in P(i)}\log p_{ij}.$$

where ϵ is a small positive constant introduced for numerical stability. This loss encourages samples belonging to the same category to form compact clusters in the latent space while increasing the separation between samples from different categories. The adjacent contrastive loss $L_{A}$ and the non-adjacent contrastive loss $L_{NA}$ are calculated in the same way as $L_{O}$.

### 3.6. Alignment Module

To address the challenge that samples from the same class are difficult to align during feature contrast, this paper proposes an alignment module consisting of $G_{1}$ and $G_{2}$, which is designed as a symmetric bottleneck architecture. Specifically, this module is composed of symmetric linear layers, batch normalization layers, and ReLU activation functions. It projects the original representation z into another space to generate the projected representation $\hat{z}$. For overlapping samples, the alignment loss function is defined as

(12) $${\hat{L}}_{O}={∥G_{1}\left(Z_{1}\right)-Z_{2}∥}_{2}^{2}+{∥G_{2}\left(Z_{2}\right)-Z_{1}∥}_{2}^{2}.$$

For adjacent augmentation and non-adjacent augmentation, the alignment losses ${\hat{L}}_{A}$ and ${\hat{L}}_{NA}$ are calculated in the same manner as described above. The final loss functions are formulated as

(13) $$L_{O}=L_{O}+\lambda {\hat{L}}_{O},$$

(14) $$L_{A}=L_{A}+\lambda {\hat{L}}_{A},$$

(15) $$L_{NA}=L_{NA}+\lambda {\hat{L}}_{NA}.$$

During training, the network parameters are optimized using a joint loss function composed of the adjacent contrastive loss $L_{A}$, the overlapping loss $L_{O}$, and the non-adjacent loss $L_{NA}$.

### 3.7. Classification

To address person identification, a feedforward neural network-based classifier is developed, integrating an encoder with a residual architecture. The overall design consists of two essential parts: a feature extraction component and a residual-based classifier. The feature extraction component employs the encoder $E_{1}$ introduced previously. For the residual classification part, the input is first processed by a linear transformation and a ReLU activation, then passed through a residual block, and finally used to classify test samples after fine-tuning. The key benefit of this design is the use of skip connections, which helps mitigate the vanishing gradient issue, enables more direct information flow, and consequently boosts both training efficiency and classification accuracy.

## 4. Experiments

To comprehensively evaluate the proposed method for EEG-based person identification, experiments were conducted from two complementary perspectives: training-data availability and cross-session robustness. For training-data availability, two sample-size conditions, namely sufficient-sample and limited-sample settings, were evaluated on the PhysioNet dataset. In addition, a cross-session evaluation was conducted on the SEED-IV dataset to investigate whether the learned identity-related representations could remain discriminative across recording sessions acquired on different days and under different emotional stimuli.

The first condition corresponded to a sufficient-sample scenario, in which the models were trained using all available training samples from each subject. This setting was designed to examine the identification capability of different methods when adequate subject-specific EEG data were available for enrollment. The evaluation was conducted on the four EEG paradigms defined in this study, including eyes-closed resting state (EC), eyes-open resting state (EO), physical motor execution (PHY), and motor imagery (IMA).

The second condition was designed to evaluate a data-constrained enrollment scenario in which only a short duration of EEG data is available for user enrollment. In this setting, only 10 one-second EEG samples from each subject were used for model training, while the remaining test data were used for performance evaluation. This experiment was intended to determine whether the proposed model could still learn discriminative identity-related representations when only limited subject-specific calibration data were available.

In addition to the above sample-size experiments, a cross-session person identification experiment was conducted on the SEED-IV dataset to further evaluate the robustness of the proposed method against session variability. The three recording sessions of SEED-IV were denoted as S1, S2, and S3. In each cross-session configuration, EEG recordings from two sessions were used as the source data for model training, while the remaining session was treated as an unseen target session and used exclusively for performance evaluation. Specifically, three configurations were considered: S1 + S2 → S3, S1 + S3 → S2, and S2 + S3 → S1. The same 15 subjects were present in all three sessions; therefore, the identification classes remained unchanged between the training and test sets, while the recording sessions differed. No EEG samples from the target session were included in the training data. The identification accuracies obtained from the three configurations were finally averaged to provide an overall measure of cross-session performance. Since the three sessions were recorded on different days and employed different emotional film stimuli, this protocol provides a challenging evaluation of whether identity-related EEG representations learned from the source sessions can generalize to an unseen recording session despite session-dependent and stimulus-related variations. For the SEED-IV cross-session experiments, non-adjacent positive samples were constructed from two sources: temporally distant segments within the same source session and segments from the other source session of the same subject. In contrast, overlapping and adjacent positive samples were generated within the corresponding source session. This design allows the model to incorporate both within-session temporal diversity and cross-session intra-subject variability during representation learning, while the held-out target session remains completely unseen during training.

In addition to identification accuracy, a cross-session representation-distance analysis was conducted to further examine whether the learned representations preserved subject-specific information across recording sessions. For each cross-session configuration, the encoder trained exclusively on the two source sessions was frozen and used to extract latent representations from both the source and held-out target sessions.

For each subject *i*, the latent representations of all samples from the two source sessions were averaged to obtain a subject-specific source reference representation:

(16) $$c_{i}^{\mathrm{src}}=\frac{1}{N_{i}^{\mathrm{src}}}\sum_{n=1}^{N_{i}^{\mathrm{src}}}z_{i,n}^{\mathrm{src}},$$

where $z_{i,n}^{\mathrm{src}}$ denotes the latent representation of the *n*-th source-session sample from subject *i*, and $N_{i}^{\mathrm{src}}$ denotes the number of source-session samples for that subject.

The cosine distance between a target-session representation z and a source reference representation c was calculated as

(17) $$d_{\cos}\left(z,c\right)=1-\frac{z^{T}c}{{∥z∥}_{2}{∥c∥}_{2}}.$$

For each target-session sample, the cosine distance to the source reference representation of the same subject was considered the same-subject cross-session distance. Accordingly, the mean same-subject distance for subject *i* was calculated as

(18) $$D_{i}^{\mathrm{same}}=\frac{1}{N_{i}^{\mathrm{tgt}}}\sum_{n=1}^{N_{i}^{\mathrm{tgt}}}d_{\cos}\left(z_{i,n}^{\mathrm{tgt}},c_{i}^{\mathrm{src}}\right).$$

In contrast, the different-subject distance was calculated by comparing each target-session sample with the source reference representations of all other subjects:

(19) $$D_{i}^{\mathrm{diff}}=\frac{1}{N_{i}^{\mathrm{tgt}}\left(C-1\right)}\sum_{n=1}^{N_{i}^{\mathrm{tgt}}}\sum_{\begin{array}{c} j=1\\ j\neq i \end{array}}^{C}d_{\cos}\left(z_{i,n}^{\mathrm{tgt}},c_{j}^{\mathrm{src}}\right),$$

where C=15 is the number of subjects in SEED-IV. Therefore, each subject contributed one same-subject distance and one different-subject distance in each cross-session configuration. A paired Wilcoxon signed-rank test was used to determine whether the same-subject cross-session distances were significantly smaller than the corresponding different-subject distances.

All experiments were implemented on an NVIDIA GeForce RTX 3090 GPU. For the experiments on the PhysioNet dataset, the continuous EEG recordings of each subject were chronologically divided into training and test sets at a ratio of 80% and 20%, respectively. Five-fold cross-validation was performed exclusively on the training set for model selection and hyperparameter tuning, while the held-out 20% test set was used only for final performance evaluation. For the cross-session experiments on SEED-IV, the two source sessions were used for model training, whereas the target session was completely held out for testing according to the three cross-session configurations described above. The models were optimized using the Adam optimizer with a learning rate of $1\times 10^{-4}$ and a batch size of 32.

## 5. Results and Discussion

Firstly, we compared TIB-MPCL with popular EEG-based person identification methods, including Fuzzy entropy [12], EEGNet [36], AITST [37], CLT [38]. Additionally, a CNN model using raw EEG signals (“Raw + CNN” [39]) was included for comparison.

The compared methods are briefly described as follows:

- Fuzzy Entropy. This method extracts fuzzy entropy features from EEG signals to describe the nonlinear complexity of each EEG segment. The extracted fuzzy entropy features are then fed into a support vector machine (SVM) classifier for subject identification.
- EEGNet. EEGNet takes EEG segments as input and learns discriminative temporal and spatial features through a compact convolutional neural network. The learned representations are then fed into the classification layer to predict the subject identity.
- AITST. AITST adopts an affective interrelated temporal–spatial Transformer framework for EEG-based person identification. It models temporal dependencies and spatial channel relationships in EEG signals to learn identity-related representations.
- CLT. CLT extracts PSD features from EEG signals and constructs positive and negative sample pairs for contrastive representation learning. Its main framework consists of an encoder with attention and Transformer modules, an MLP projection head for contrastive learning, and a fine-tuned fully connected layer for final identity classification.
- Raw + CNN. The Raw + CNN baseline takes raw EEG time-series segments as input to a convolutional neural network. The CNN automatically learns subject-discriminative representations from the time-series signals and performs identity classification through its final classification layer.

### 5.1. Training with Sufficient Samples

We evaluated the proposed model under a scenario with sufficient training samples. The number of training epochs was set to 100 for the contrastive learning stage and 20 for the fine-tuning stage. As shown in Table 1, the proposed TIB-MPCL achieves highly competitive performance under the sufficient-sample condition. Compared with other algorithms, TIB-MPCL achieves the highest average accuracy and shows consistently competitive performance across the four paradigms. Specifically, Fuzzy Entropy and EEGNet obtain average accuracies of 80.46% and 84.36%, respectively, while Raw + CNN improves the average accuracy to 89.97%. In contrast, methods based on Transformer achieve substantially higher accuracies. AITST and CLT obtain average accuracies of 97.89% and 99.00%, respectively, demonstrating the effectiveness of learning discriminative temporal features from EEG signals. The proposed TIB-MPCL further improves the average accuracy to 99.05%, achieving the best overall performance among all compared methods. In particular, TIB-MPCL obtains the highest accuracy in the EO, PHY, and IMA paradigms, with accuracies of 98.86%, 99.38%, and 99.62%, respectively. Although CLT performs better in the EC paradigm, TIB-MPCL maintains consistently high accuracy across all paradigms, with all results above 98%. These results suggest that the proposed method can effectively learn robust and discriminative EEG representations when sufficient training samples are available. The performance gains over handcrafted feature-based and CNN-based baselines further indicate the effectiveness of contrastive representation learning for EEG-based person identification. Overall, TIB-MPCL demonstrates strong identification capability and good generalization across different EEG paradigms under sufficient-sample conditions.

### 5.2. Training with Limited Samples

To evaluate the robustness of the proposed TIB-MPCL framework under data-constrained conditions, we further conducted experiments in a limited-sample training scenario. In this setting, only 10 one-second EEG samples per subject were used for enrollment and model training, while the remaining data were reserved for testing. This configuration evaluates data-efficient subject identification under a within-session limited-enrollment setting.

The experimental results are summarized in Table 2. Overall, all comparison methods exhibit substantial performance degradation under the limited-sample condition. Fuzzy Entropy, EEGNet, and Raw + CNN achieve average accuracies of only 12.48%, 14.87%, and 22.08%, respectively. Their identification accuracies across the four paradigms range from 10.10% to 26.69%. More severely, AITST and CLT yield near-random performance, with average accuracies of 1.27% and 3.34%, respectively. These results indicate that conventional feature-based, convolutional, and Transformer-based methods rely heavily on sufficient training data and are susceptible to severe overfitting when only a small number of enrollment samples are available.

In contrast, the proposed TIB-MPCL demonstrates strong robustness under the same limited-sample condition. It achieves identification accuracies of 79.02%, 73.99%, 91.28%, and 91.14% on the EO, EC, PHY, and IMA paradigms, respectively, resulting in an average accuracy of 83.86%. Compared with the best-performing baseline in each paradigm, TIB-MPCL improves the accuracy by 58.18, 53.30, 71.20, and 64.45 percentage points, respectively. Notably, the proposed model maintains accuracies above 91% on both the motor execution (PHY) and motor imagery (IMA) paradigms, substantially outperforming all comparison methods. These results demonstrate that the proposed time-interval-based multi-positive contrastive learning strategy can effectively extract discriminative identity-related representations even when only a small number of training samples are available.

The superior performance of TIB-MPCL can be attributed to two key factors. First, the proposed multi-positive augmentation strategy (including overlapping, adjacent, and non-adjacent temporal segments) enhances intra-class compactness by enforcing consistency across temporally correlated EEG segments. This alleviates the instability caused by limited training data. Second, the supervised contrastive learning objective explicitly maximizes inter-class separation while minimizing intra-class variance in the embedding space, which is particularly beneficial under small-sample conditions compared to conventional cross-entropy-based classification.

It is also observed that performance under the resting-state paradigms (EO and EC) is relatively lower than that under the task-related paradigms (PHY and IMA), particularly in the limited-sample setting. Resting-state EEG has practical advantages for biometric enrollment because it requires little or no explicit task engagement; however, its spontaneous neural activity generally exhibits greater temporal variability and weaker task-locked structure, which may make subject-discriminative patterns more difficult to capture from only a few enrollment samples. In contrast, motor execution and motor imagery paradigms induce more structured and repeatable neural responses, which may provide more stable identity-related representations and partly explain the higher identification accuracies observed in PHY and IMA.

Beyond resting-state and motor-related paradigms, event-related EEG responses have also been investigated for biometric identification. Stimulus-locked responses, such as event-related potentials, can provide reproducible temporal patterns that may contain subject-specific information, although such approaches generally require controlled stimulus presentation and may increase enrollment complexity. The proposed TIB-MPCL framework is not intrinsically restricted to motor-related EEG because its positive samples are constructed according to temporal relationships between EEG segments rather than task-specific signal characteristics. Accordingly, the multi-positive contrastive learning strategy may potentially be extended to other EEG elicitation paradigms, including event-related biometric protocols. Nevertheless, the current experiments do not directly evaluate dedicated event-related potential datasets. Therefore, further validation on ERP-based and other task-independent EEG biometric datasets will be necessary to establish the generalizability of TIB-MPCL across different acquisition and elicitation paradigms.

Overall, these results demonstrate that TIB-MPCL maintains strong performance even under severely limited training conditions, significantly reducing the reliance on large-scale enrollment data. These findings suggest the potential of the proposed method for data-efficient EEG biometric systems.

### 5.3. Effect of the Number of Training Samples per Subject

**Figure 3:**
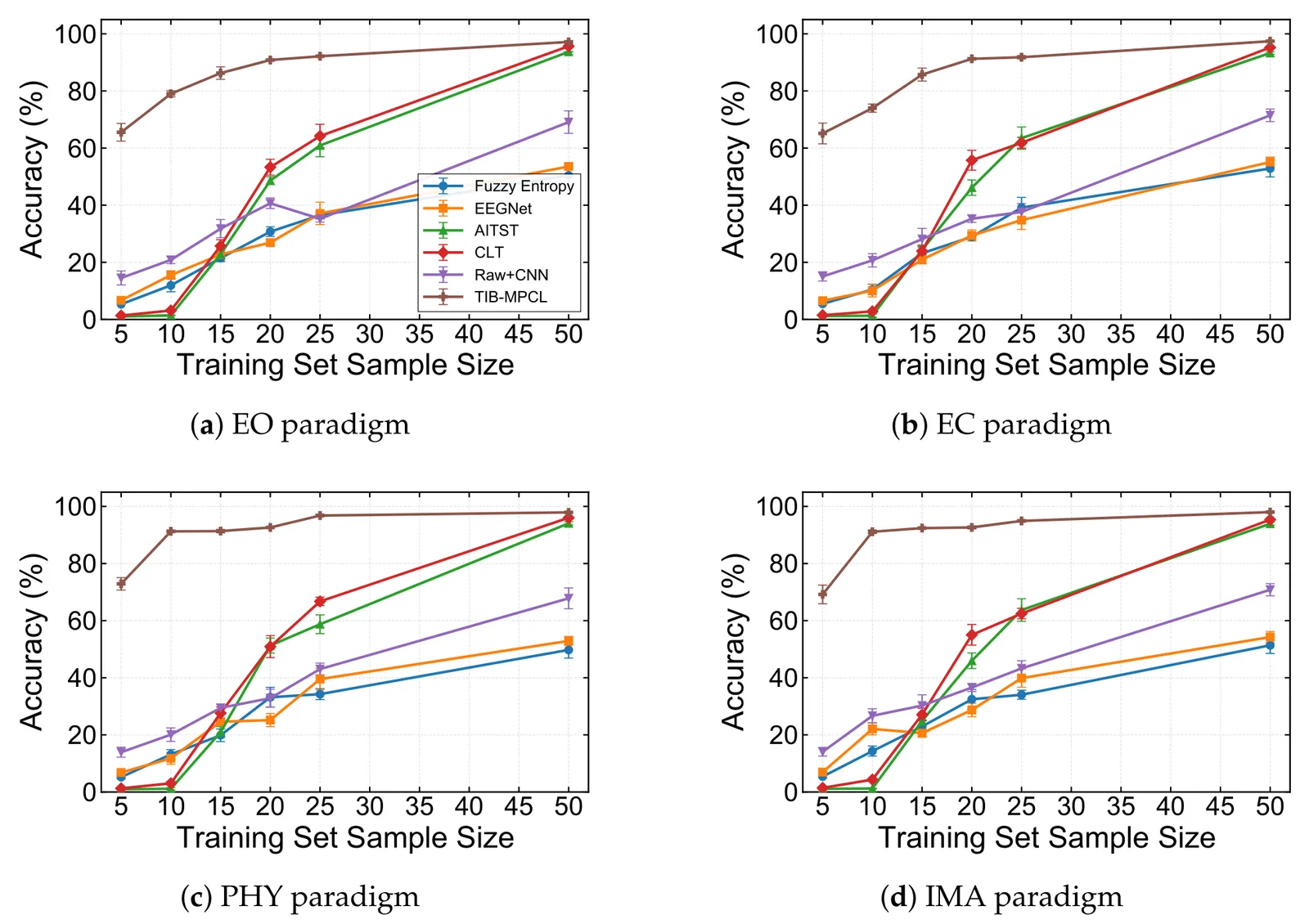
Effect of the number of training samples per subject on recognition accuracy under different paradigms.

To investigate the influence of the number of training samples per subject, we varied the number of samples selected from each subject as [5,10,15,20,25,50] and evaluated all methods under the EO, EC, PHY, and IMA paradigms. Overall, increasing the number of training samples consistently improves the recognition accuracy for most methods, indicating that additional subject-specific samples provide more discriminative information for model training. However, different algorithms exhibit different degrees of sensitivity to the sample size. As shown in Figure 3, TIB-MPCL consistently achieves the best performance under all four paradigms.

For traditional feature-based methods such as Fuzzy Entropy and EEGNet, the accuracy remains relatively low when only 5 or 10 samples are used. Averaged over the four paradigms, Fuzzy Entropy improves from 5.36% with 5 samples to 51.11% with 50 samples, while EEGNet improves from 6.76% to 53.96%. Although their performance increases as more samples are introduced, the final accuracy is still much lower than that of the stronger learning-based methods, suggesting that these methods have limited ability to exploit the additional training data.

AITST and CLT show a much stronger dependence on the number of training samples. When the sample size is small, their performance is very limited, with average accuracies of only 1.08% and 1.39% at 5 samples, respectively. Their performance increases sharply after the number of samples reaches 15 or 20. At 50 samples, AITST and CLT achieve average accuracies of 93.77% and 95.59%, respectively. This indicates that these methods require sufficient training samples to learn effective representations, but can achieve competitive performance when adequate data are available.

The raw+CNN method shows a more gradual improvement. Its average accuracy increases from 14.40% at 5 samples to 69.80% at 50 samples. Compared with AITST and CLT, raw+CNN performs better in the extremely low-sample setting, but its final performance with 50 samples is still substantially lower.

In contrast, the proposed TIB-MPCL method achieves consistently high accuracy across all sample sizes and paradigms. Even when only 5 samples per subject are used, TIB-MPCL obtains an average accuracy of 68.18%, clearly outperforming all comparison methods. As the number of samples increases, its accuracy further improves and reaches 97.64% on average with 50 samples. Moreover, TIB-MPCL already achieves over 90% accuracy in most paradigms when 20 or 25 samples are available, demonstrating its robustness under limited training data. These results show that TIB-MPCL is less sensitive to the number of training samples and can effectively learn discriminative representations even in low-sample scenarios.

### 5.4. Authentication Performance Based on ROC, EER, and TAR Analysis

In addition to identification accuracy, we further evaluated the proposed TIB-MPCL model from the perspective of biometric authentication under the EO paradigm. Specifically, the receiver operating characteristic (ROC) curve, area under the curve (AUC), equal error rate (EER), and true acceptance rates (TARs) at predefined false acceptance rates (FARs) were calculated under different numbers of training samples per subject. TAR represents the proportion of genuine authentication attempts that are correctly accepted at a specified FAR, with a higher TAR indicating better authentication performance.

Table 3 summarizes the authentication results under different sample-size settings. Overall, the proposed method achieves consistently high AUC values and low EER values across all settings. When only five samples per subject are used for training, the model already obtains an AUC of 98.13% and an EER of 6.68%, indicating that the learned EEG representations retain strong discriminative ability even under an extremely limited enrollment condition. As the number of training samples increases, the authentication performance improves steadily. Specifically, the EER decreases from 6.68% with 5 samples to 1.24% with 50 samples, while the AUC increases from 98.13% to 99.88%.

In addition, the TARs at FARs of 1% and 0.1% generally improve as more training samples are introduced. When all available training samples are used, the TAR reaches 99.49% at FAR = 1% and 98.86% at FAR = 0.1%, indicating that the model can correctly accept the vast majority of genuine users even under stringent false-acceptance constraints.

**Figure 4:**
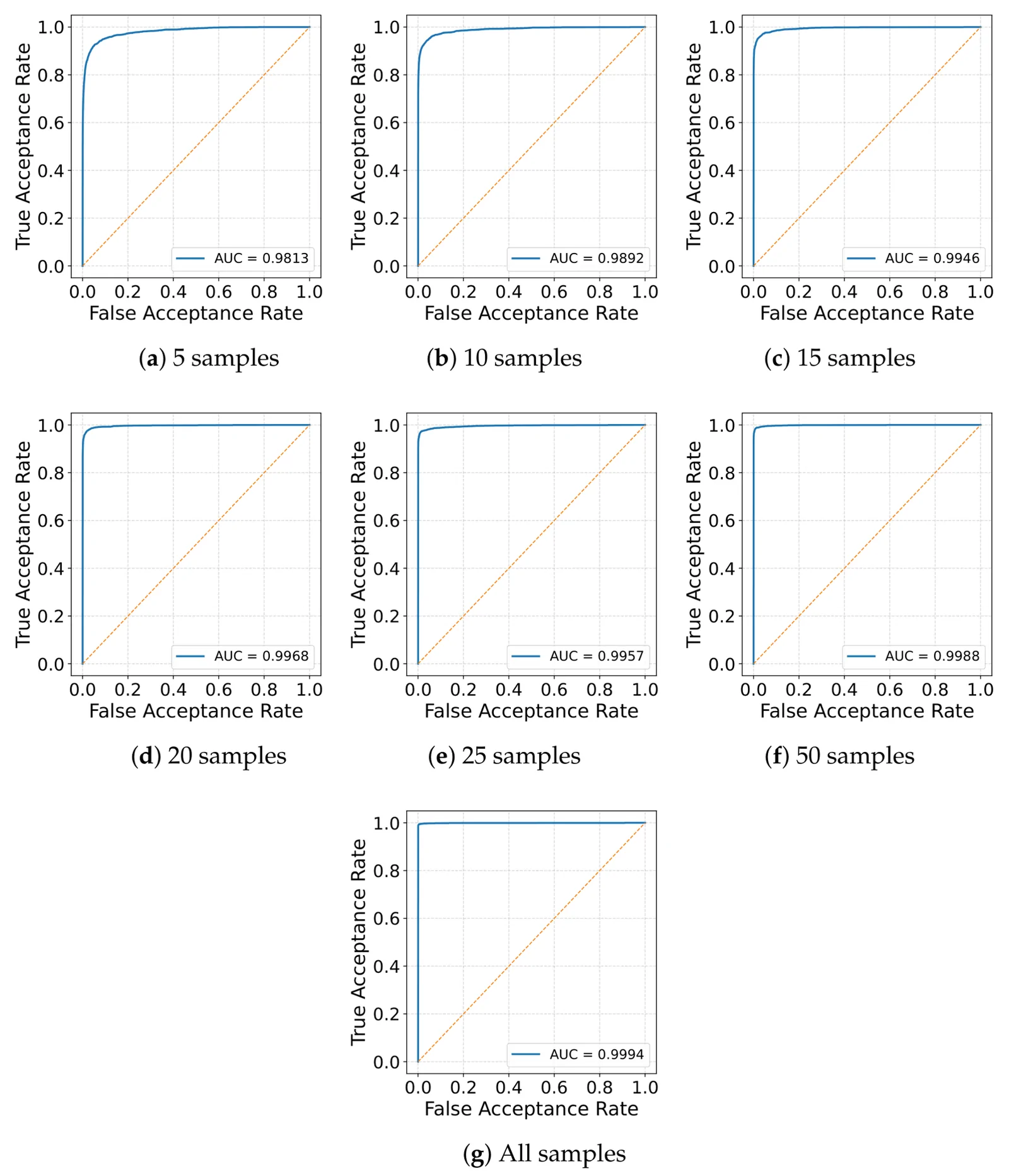
ROC curves for EEG-based biometric authentication under the EO paradigm with different numbers of training samples per subject. The dashed diagonal line represents the performance of a random classifier.

**Figure 5:**
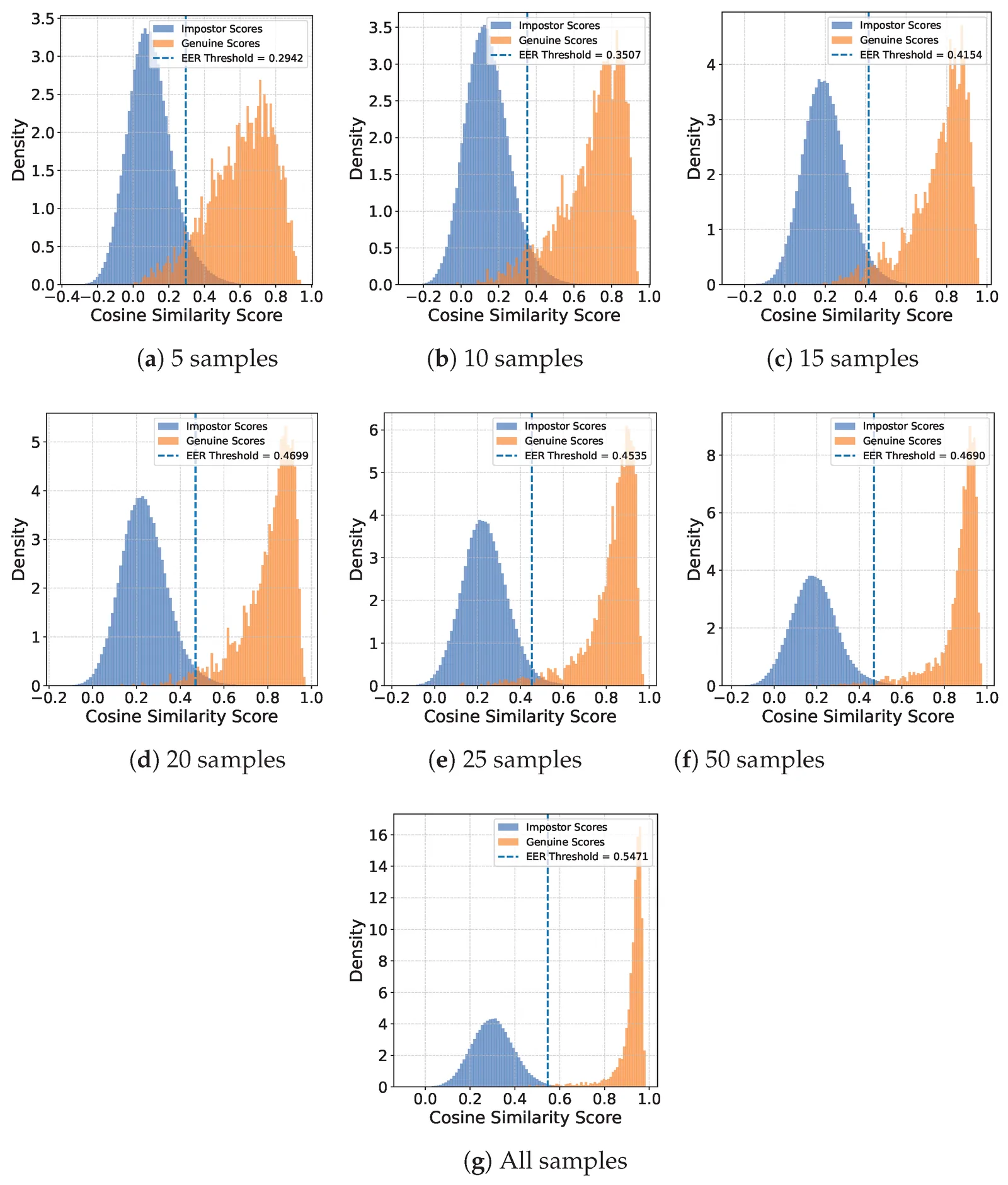
Genuine and impostor score distributions for EEG-based biometric authentication under the EO paradigm with different numbers of training samples per subject.

Furthermore, ROC curves were generated for different sample-size settings to provide a more comprehensive visualization of the authentication performance, as shown in Figure 4. In addition, the Genuine and Impostor Score Distributions were plotted for each training sample-size setting to further examine the separability of authentication scores, as shown in Figure 5. In biometric authentication, genuine scores are obtained from matching samples belonging to the same subject, while impostor scores are obtained from matching samples belonging to different subjects. Therefore, the degree of separation between the two distributions directly reflects the discriminative capability of the learned subject-specific representations. Ideally, genuine scores should be concentrated in a relatively high-similarity region, whereas impostor scores should remain in a low-similarity region, leading to a clear decision boundary between the two distributions.

From the plotted score distributions, it can be observed that the genuine and impostor distributions are already distinguishable even when only a small number of training samples are available. This indicates that the proposed TIB-MPCL model can learn effective biometric representations under limited-sample conditions. However, the corresponding EER threshold is located at a relatively lower cosine similarity value when the number of training samples is small. For example, when only 5 samples per subject are used, the EER threshold is 0.2942. As the number of training samples increases, the EER threshold gradually shifts toward a higher cosine similarity region, increasing to 0.4690 with 50 samples and further reaching 0.5471 when all available samples are used.

As the number of training samples increases, the genuine-score distribution shifts toward higher similarity values and becomes increasingly separated from the impostor-score distribution. Correspondingly, the EER decreases and the TAR increases, indicating improved authentication discriminability.

### 5.5. Ablation Experiments

To further investigate the effectiveness of each component in the proposed method, we conduct an ablation study under the EO paradigm. Specifically, we remove or replace different modules and evaluate the corresponding performance under different numbers of training samples. The results are reported in Table 4. The columns 5, 10, 15, 20, 25, 50, and all samples denote the number of training samples used in the EO setting.

As shown in Table 4, the proposed method consistently achieves the best performance across all training sample settings. Compared with all ablated variants, our method obtains accuracies of 65.53%, 79.02%, 86.26%, 90.85%, 92.17%, 97.17%, and 98.86% when using 5, 10, 15, 20, 25, 50, and all training samples, respectively. These results demonstrate that the complete framework is effective and that each component contributes positively to the final performance.

When the alignment module is removed, performance decreases across all sample settings. For example, the accuracy drops from 86.26% to 83.37% with 15 training samples and from 90.85% to 88.31% with 20 training samples. This indicates that the alignment module plays an important role in learning more consistent and discriminative representations. By aligning feature representations, the model can better reduce distribution discrepancies among samples, thereby improving overall recognition performance.

The AR+ENCODER variant also shows clear performance degradation compared with the proposed method. In particular, accuracy decreases from 79.02% to 74.09% with 10 training samples, resulting in a performance drop of 4.93%. Similar decreases can also be observed under other training sample settings. These results suggest that simply using AR+ENCODER is insufficient to fully capture the discriminative relationships among samples. In contrast, the proposed framework can better exploit the structural relationships in the EO paradigm and thus achieves better performance.

We further compare the effects of different types of positive sample pairs, including overlapping positive sample pairs, adjacent positive sample pairs, and non-adjacent positive sample pairs. When the overlapping positive sample pairs are removed, the performance decreases from 65.53% to 64.93% with 5 training samples, and from 98.86% to 97.75% when all samples are used. Although the performance degradation is relatively moderate, the consistent decrease across different sample settings indicates that overlapping positive sample pairs provide useful local consistency constraints for representation learning.

When the adjacent positive sample pairs are removed, the performance drops more significantly, especially in low-sample scenarios. For example, the accuracy decreases from 65.53% to 62.55% with 5 training samples, and from 79.02% to 76.00% with 10 training samples. These decreases of 2.98% and 3.02%, respectively, suggest that adjacent positive sample pairs are particularly important when the available training samples are limited. This is because adjacent positive pairs can provide strong local relational information, which helps the model learn more robust representations under the EO paradigm.

In contrast, removing non-adjacent positive sample pairs leads to more obvious performance degradation in the medium-sample settings. For instance, the accuracy decreases from 86.26% to 83.67% with 15 training samples, from 90.85% to 87.90% with 20 training samples, and from 92.17% to 90.22% with 25 training samples. These results suggest that non-adjacent positive sample pairs provide complementary information from temporally more distant EEG segments. This observation is consistent with the hypothesis that incorporating longer-range temporal diversity can help improve the discriminative quality of the learned representations.

We further evaluated the contribution of ICA preprocessing by removing ICA while keeping the remaining framework unchanged. Removing ICA resulted in a modest overall decrease in recognition accuracy, indicating that ICA provides a beneficial preprocessing effect for the proposed framework. Nevertheless, the model maintained high identification performance without ICA, suggesting that the observed subject-discriminative information is not solely dependent on ICA preprocessing. These results indicate that ICA provides a beneficial preprocessing effect while not being essential for the identification capability of the proposed method.

Overall, the comparison among the three types of positive sample pairs shows that they play complementary roles. Overlapping positive sample pairs help preserve local consistency, adjacent positive sample pairs are more critical in extremely low-sample scenarios, while non-adjacent positive sample pairs introduce complementary information from temporally more distant EEG segments. The superior performance of the proposed method verifies the effectiveness of jointly incorporating these positive sample pairs under the EO paradigm.

### 5.6. Comparison of Electrode Selection

**Figure 6:**
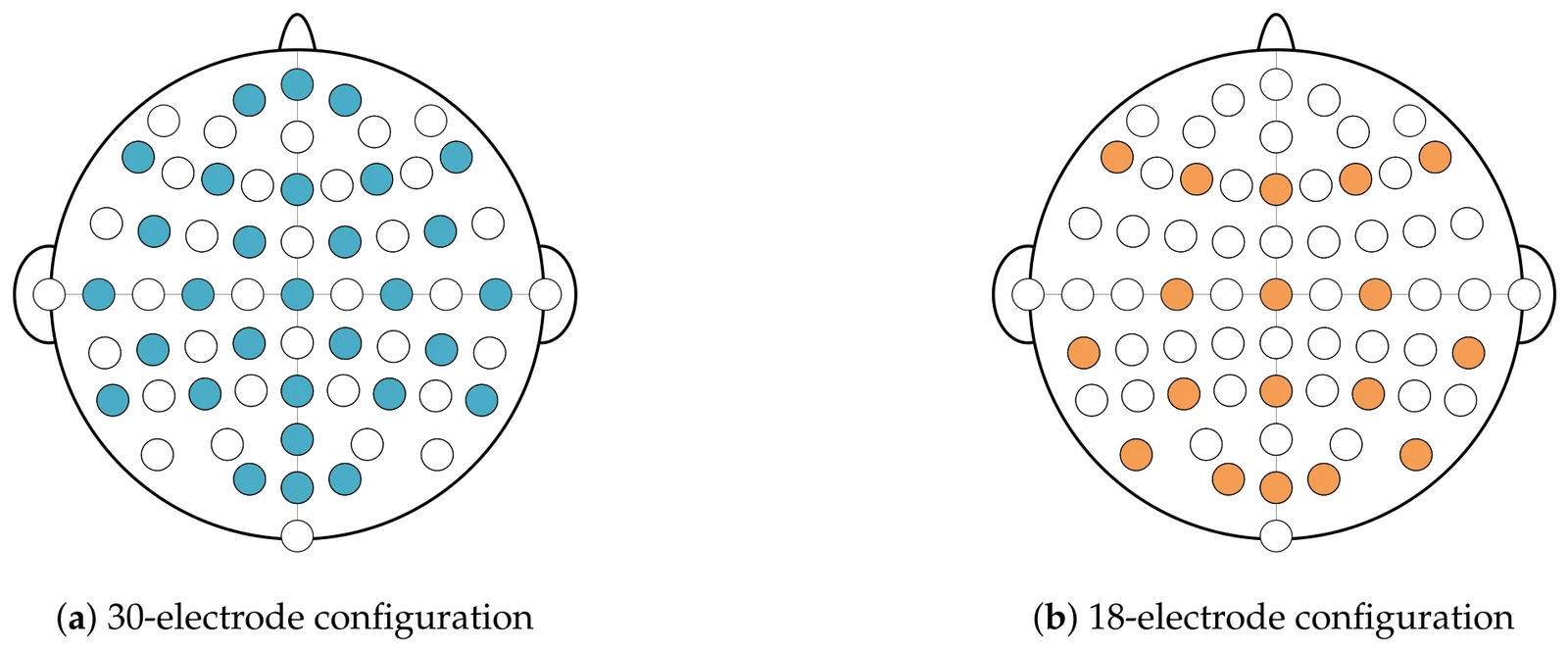
Spatial distributions of the selected electrodes under the 30-electrode and 18-electrode configurations. Blue and orange circles denote the selected electrodes for the 30-electrode and 18-electrode configurations, respectively.

To further investigate the influence of electrode number on identification performance, additional experiments were conducted on the PhysioNet dataset under the sufficient-sample setting. In addition to the original 64-channel configuration, two reduced electrode configurations containing 30 and 18 electrodes were evaluated, with the electrode selection referring to the spatial configurations adopted in previous study [40]. The corresponding electrode distributions are illustrated in Figure 6, and the identification results are summarized in Table 5. As the number of electrodes decreases, the identification accuracy shows a gradual decline across all four paradigms. The 64-electrode configuration achieves an average accuracy of 99.05%, while the 30- and 18-electrode configurations achieve 97.66% and 93.73%, respectively.

Notably, reducing the number of electrodes from 64 to 30 results in only a moderate performance decrease, with accuracies remaining above 96% for all four paradigms. Even with only 18 electrodes, TIB-MPCL maintains accuracies above 93% across all paradigms. These results indicate that although a denser electrode configuration provides more comprehensive spatial information, the proposed method can still achieve competitive identification performance with substantially fewer electrodes, suggesting its potential for EEG biometric systems with reduced electrode configurations.

### 5.7. Cross-Session Person Identification

To further evaluate the robustness of TIB-MPCL to session-dependent variations, cross-session person identification experiments were conducted on the SEED-IV dataset. As shown in Table 6, TIB-MPCL achieved accuracies of 68.32%, 97.43%, and 85.61% under the S2 + S3 → S1, S1 + S3 → S2, and S1 + S2 → S3 settings, respectively, with an average accuracy of 83.79%. TIB-MPCL achieved the best performance in two of the three settings and remained comparable to CLT in the S1 + S3 → S2 setting.

To assess whether the cross-session performance depends on ICA preprocessing, we repeated the experiments without ICA while keeping all other settings unchanged. The corresponding accuracies were 63.27%, 95.31%, and 83.02%, with an average of 80.53%, representing an average decrease of 3.26 percentage points. The model therefore retained substantial cross-session identification capability without ICA, suggesting that the observed subject-discriminative information is not solely attributable to ICA preprocessing or subject/session-specific ICA transformations.

**Figure 7:**
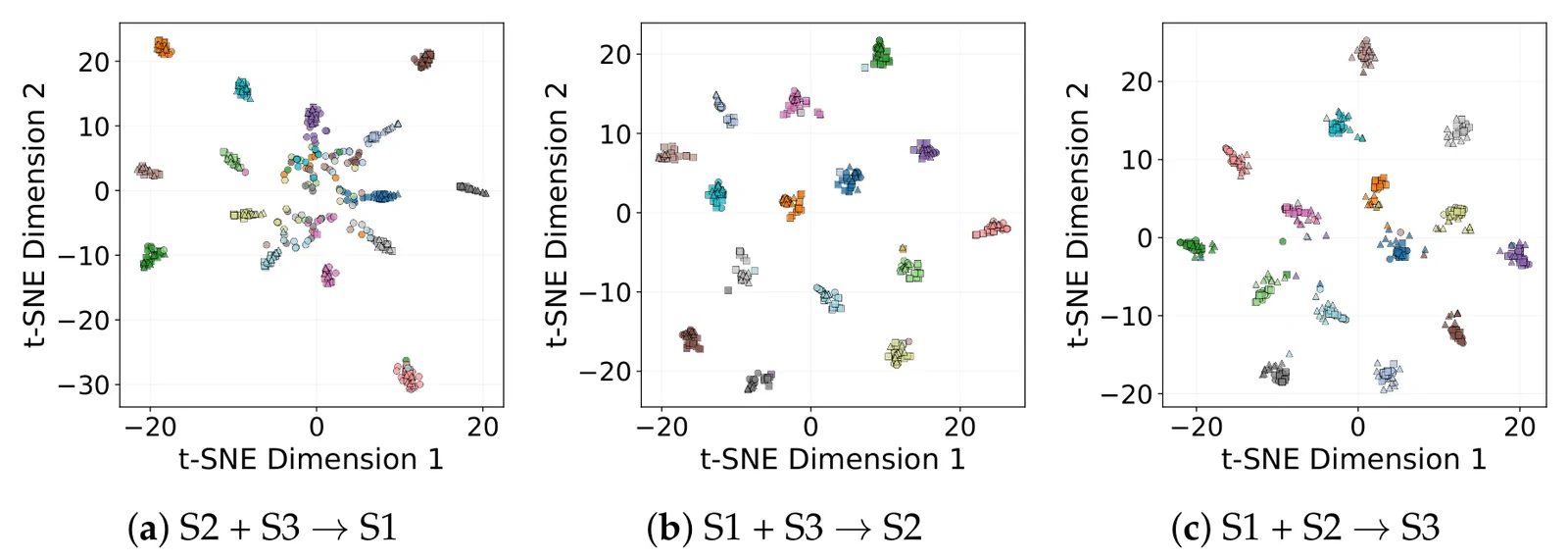
t-SNE visualization of the representations learned by TIB-MPCL under three cross-session person identification settings on the SEED-IV dataset. Colors indicate different subjects, and marker shapes indicate different recording sessions (S1: circle; S2: square; S3: triangle).

Figure 7 further visualizes the learned representations across the three cross-session settings. Samples from the same subject tend to form relatively compact clusters across sessions, although some inter-subject overlap remains, supporting the presence of cross-session subject-discriminative structure in the learned representation space.

Taken together, the quantitative and visualization results suggest that the proposed method can maintain discriminative subject-related representations across different recording sessions. Since the three SEED-IV sessions were collected on different days and under different emotional stimuli, the results also indicate that the time-interval-based multi-positive contrastive learning strategy provides a certain degree of robustness to session-dependent variability. Overall, this experiment further supports the generalization capability of TIB-MPCL beyond the within-session evaluation on PhysioNet.

The considerable performance variation across target sessions also indicates that session-dependent distribution shifts remain non-negligible. In particular, the lower performance when S1 is used as the unseen target suggests that cross-session variability is not fully eliminated by the proposed representation. Therefore, although TIB-MPCL improves cross-session robustness relative to the baselines, session invariance remains an open challenge.

To further examine the cross-session stability of subject-discriminative information, we compared the cosine distances between the held-out target-session samples and the source-session reference representations in both the original AR feature space and the learned representation space. Specifically, the same-subject distance measures the distance between a target-session sample and the source reference representation of the same subject, whereas the different-subject distance measures its average distance to the reference representations of the remaining subjects.

As shown in Table 7, the mean same-subject cosine distances are lower than the mean different-subject distances in the original AR feature space. Paired Wilcoxon signed-rank tests confirmed significant differences in all three configurations (p<0.001 for each configuration), indicating that the input AR features already retain a certain degree of cross-session subject-specific information.. More importantly, the separation between same-subject and different-subject distances becomes much more pronounced in the learned representation space. Specifically, under the S2 + S3 → S1 setting, the distance margin increases from 0.0008 in the original AR feature space to 0.3638 in the learned representation space. Similar increases are observed for S1 + S3 → S2, from 0.0008 to 0.6162, and for S1 + S2 → S3, from 0.0007 to 0.4254. These results indicate that TIB-MPCL transforms the original AR features into a more discriminative representation space with greater separation between same-subject and different-subject cross-session relationships.

**Figure 8:**
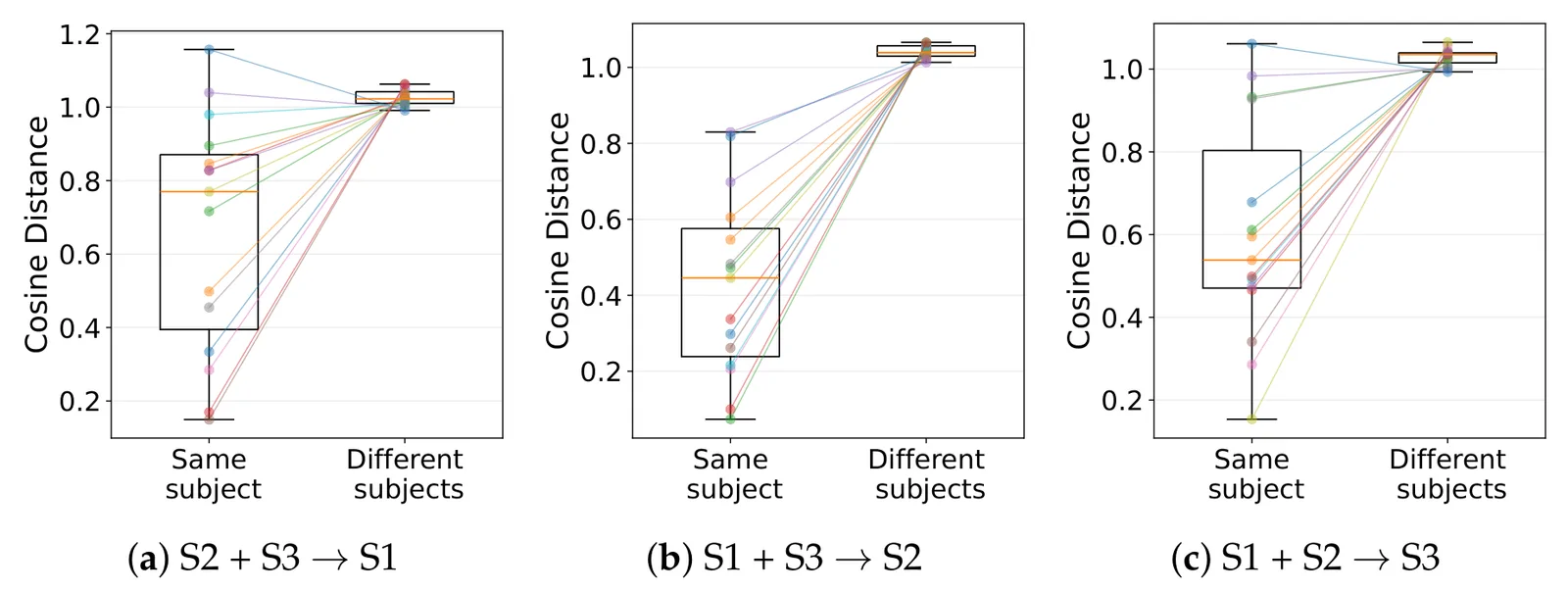
Comparison of same-subject and different-subject cosine distances across recording sessions on the SEED-IV dataset. For each cross-session configuration, the same-subject distance was calculated between target-session embeddings and the corresponding source-session subject reference, whereas the different-subject distance was calculated with respect to the reference representations of the other subjects. Lower cosine distance indicates greater similarity in the learned representation space. Different colors represent different subjects, and the connecting lines link the corresponding same-subject and different-subject distances.

As further illustrated in Figure 8, the same-subject cross-session distances were, on average, smaller than the different-subject distances across all three configurations. Specifically, the mean same-subject and different-subject distances were 0.6634 and 1.0272 for S2 + S3 → S1, 0.4260 and 1.0422 for S1 + S3 → S2, and 0.6036 and 1.0290 for S1 + S2 → S3, respectively. Paired Wilcoxon signed-rank tests showed significant differences between the same-subject and different-subject distances in all three configurations (p<0.001 for each configuration). These results indicate an overall tendency for the learned representations to preserve greater cross-session similarity within subjects than between different subjects.

### 5.8. Limitations and Future Directions

Despite the encouraging results, several limitations should be acknowledged. First, the PhysioNet experiments use a within-session chronological split and therefore primarily evaluate data-efficient person identification under a shared acquisition context. These experiments alone cannot establish long-term biometric permanence. Second, although the SEED-IV evaluation provides cross-session testing using recordings acquired on different days, SEED-IV was originally designed for emotion recognition rather than biometric identification, and includes only 15 subjects. Moreover, detailed acquisition factors such as electrode impedance, exact electrode repositioning, and device variability are not explicitly controlled in the present study. Although the cross-session results indicate that subject-discriminative information persists across recording days and emotional conditions, they should not be interpreted as evidence that the identified signatures are exclusively neuronal in origin. Scalp EEG reflects not only neural activity but may also be influenced by residual ocular and muscular activity, subject-specific anatomical properties, tissue conductivity, and electrode–scalp coupling. ICA was employed to attenuate major artifacts, but complete elimination of non-neural contributions cannot be guaranteed. Third, the proposed overlapping and adjacent augmentations operate within individual recording sessions and are intended to improve representation learning under limited enrollment data rather than to simulate independent acquisition sessions. Future work should therefore evaluate TIB-MPCL on dedicated longitudinal EEG biometric datasets involving larger populations, repeated electrode removal and reapplication, longer inter-session intervals, different acquisition devices, and single-session enrollment followed by independent-session re-identification. In addition, dedicated EOG/EMG recordings, stricter artifact-control procedures, and physiological feature analyses should be incorporated to better disentangle neural and non-neural sources of subject specificity.

## 6. Conclusions

In this study, we proposed a data-efficient EEG-based person identification framework named Time-Interval-Based Multi-Positive Contrastive Learning (TIB-MPCL). The proposed method aims to reduce the dependence of EEG-based biometric systems on large-scale enrollment data. By extracting AR-based temporal features from EEG signals and constructing overlapping, adjacent, and non-adjacent positive sample pairs, TIB-MPCL effectively exploits the temporal relationships among EEG segments. In addition, the supervised contrastive learning strategy and alignment module encourage intra-subject compactness and inter-subject separability in the latent representation space, enabling the model to learn discriminative identity-related features even when only a small number of training samples are available.

Extensive experiments on the PhysioNet EEG dataset involving 109 subjects demonstrate the effectiveness of the proposed method across four paradigms, including EO, EC, PHY, and IMA. Under the sufficient-sample condition, TIB-MPCL achieves an average identification accuracy of 99.05%, showing highly competitive performance compared with existing EEG-based identification methods. More importantly, under the limited-sample condition with only 10 one-second EEG samples per subject, the proposed method achieves an average accuracy of 83.86%, substantially outperforming traditional feature-based methods, CNN-based models, and Transformer-based baselines. Further analysis shows that TIB-MPCL remains robust as the number of training samples varies, achieving an average accuracy of 68.18% with only 5 samples per subject and 97.64% with 50 samples per subject.

The authentication analysis further demonstrates the discriminative capability of the proposed framework under different enrollment-data conditions. Under the EO paradigm, TIB-MPCL achieves an AUC of 98.13% and an EER of 6.68% with only 5 training samples per subject. When all available training samples are used, the AUC increases to 99.94%, and the EER decreases to 0.60%. Moreover, the model achieves TARs of 99.49% and 98.86% at FARs of 1% and 0.1%, respectively, demonstrating strong separability between genuine and impostor scores and reliable authentication performance under stringent false-acceptance constraints. The ablation experiments also verify the contribution of the alignment module and the three types of positive sample pairs. Specifically, overlapping, adjacent, and non-adjacent positive sample pairs are constructed for each anchor sample to incorporate complementary temporal information at different temporal distances.

Overall, the proposed TIB-MPCL framework provides an effective framework for small-sample EEG-based person identification and authentication. By reducing the amount of enrollment data required for model training, this method provides encouraging evidence toward more data-efficient EEG biometric systems. Furthermore, the cross-session experiments on SEED-IV show that the proposed framework maintains promising identification performance across recording sessions acquired on different days, providing additional evidence of robustness to session-dependent variability. Nevertheless, practical biometric deployment requires further validation on larger multi-session populations, with repeated electrode placement, longer inter-session intervals, and different acquisition devices. In future work, we will extend the cross-session evaluation to larger and more diverse longitudinal EEG datasets and investigate robustness under more realistic acquisition variations. Moreover, improving model efficiency and investigating real-time deployment will be important directions for advancing practical EEG-based biometric authentication systems.

## Figures and Tables

**Table 1:** Results of models trained in each paradigm with sufficient training samples. Results are average identification accuracy (%).

Methods	EO	EC	PHY	IMA
Fuzzy Entropy	82.55	80.37	79.30	79.64
EEGNet	83.76	85.67	83.73	84.27
AITST	97.49	97.81	98.93	97.34
CLT	98.73	99.34	99.17	98.76
Raw + CNN	91.84	84.31	91.70	92.03
TIB-MPCL	98.86	98.35	99.38	99.62

**Table 2:** Results of models trained in each paradigm with limited training samples. Results are average identification accuracy (%).

Methods	EO	EC	PHY	IMA
Fuzzy Entropy	11.94	10.48	13.17	14.32
EEGNet	15.52	10.10	11.78	22.08
AITST	1.42	1.30	1.15	1.22
CLT	3.13	2.82	3.05	4.34
Raw + CNN	20.84	20.69	20.08	26.69
TIB-MPCL	79.02	73.99	91.28	91.14

**Table 3:** Authentication performance of the proposed method under different numbers of training samples per subject.

Number of Samples	AUC (%)	EER (%)	TAR @ FAR = 1%	TAR @ FAR = 0.1%
5	98.13	6.68	80.71	59.79
10	98.92	4.85	89.45	77.21
15	99.46	3.49	93.09	86.03
20	99.68	2.31	96.14	89.56
25	99.57	2.39	96.77	92.72
50	99.88	1.24	98.57	95.85
All Samples	99.94	0.60	99.49	98.86

**Table 4:** Ablation study results under the EO paradigm with different numbers of training samples.

Method	5	10	15	20	25	50	All Samples
Without Alignment Module	64.86	77.91	83.37	88.31	90.99	96.06	97.64
AR + ENCODER	63.39	74.09	83.70	88.53	90.07	94.44	97.35
Without Overlapping Positive Sample Pair	64.93	77.73	86.02	89.41	91.94	95.73	97.75
Without Adjacent Positive Sample Pair	62.55	76.00	85.28	88.08	91.10	95.69	97.60
Without Non-Adjacent Positive Sample Pair	63.98	77.62	83.67	87.90	90.22	95.51	97.53
Without ICA	64.84	78.57	85.60	90.31	91.80	96.59	98.11
Our Proposed Method	65.53	79.02	86.26	90.85	92.17	97.17	98.86

**Table 5:** Identification performance of TIB-MPCL with different numbers of electrodes. Results are the average identification accuracy (%).

Electrode Number	EO	EC	PHY	IMA
64	98.86	98.35	99.38	99.62
30	97.57	96.81	97.92	98.34
18	93.52	93.45	94.65	93.28

**Table 6:** Cross-session person identification results on the SEED-IV dataset. Results are identification accuracy (%).

Methods	S2 + S3 → S1	S1 + S3 → S2	S1 + S2 → S3
Fuzzy Entropy	24.42	38.13	35.62
EEGNet	62.03	87.72	72.06
AITST	62.28	96.23	82.19
CLT	63.77	98.10	83.15
Raw + CNN	44.90	67.97	71.19
Without ICA	63.27	95.31	83.02
TIB-MPCL	68.32	97.43	85.61

**Table 7:** Cross-session cosine-distance analysis in the original AR feature space and the learned representation space on the SEED-IV dataset. Same-subject and different-subject distances were calculated between the held-out target session and the subject-specific reference representations constructed from the two source sessions.

	AR Feature Space		Learned Representation Space	
	Same-Subject	Different-Subject	Same-Subject	Different-Subject
S2 + S3 → S1	0.0033	0.0041	0.6634	1.0272
S1 + S3 → S2	0.0031	0.0039	0.4260	1.0422
S1 + S2 → S3	0.0031	0.0038	0.6036	1.0290

## Data Availability

Data and code supporting the findings of this study are available from the corresponding authors upon reasonable request.
